# Eggerthia catenaformis infection originating from a dental abscess causes severe intestinal complications and osteomyelitis of the jaw

**DOI:** 10.3205/iprs000152

**Published:** 2021-04-14

**Authors:** Andreas Sakkas, Isabel Nolte, Sebastian Heil, Boris Mayer, Steffen Kargus, Robert A. Mischkowski, Oliver C. Thiele

**Affiliations:** 1 Department of Oral, Maxillofacial and Facial Plastic Surgery, Ludwigshafen Hospital, Ludwigshafen, Germany

**Keywords:** Eggerthia catenaformis, anaerobic bacteria, dental abscess, osteomyelitis of the jaw, perihepatic abscesss

## Abstract

**Introduction:** Odontogenic foci may result to generalized infections spreading the bacteria through contiguous anatomic cavities or hematogenous spread. The most reported secondary infections caused by oral pathogens are intracranial abscesses. Although, few reports in the literature describe the bacterial spread to extracranial locations.

**Case** **description:** We describe the case of a 52-year-old male Caucasian patient who was admitted to our hospital suffering from severe sepsis caused by a submandibular abscess. *Eggerthia catenaformis* was detected in blood and abscess material (confirmed by MALDI-TOF mass spectrometry). The patient subsequently developed a perihepatic abscess and colon perforation, and was stabilized after several surgical interventions. He remained hospitalized for 66 days receiving intravenous antibiotics. Five months later, jaw osteonecrosis with Actinomyces contamination was detected in the left mandible, which also had to be treated surgically. Three years after the last surgery, no signs of recurrence have been detected.

**Discussion:** Oral and maxillofacial surgeons should understand the characteristics of systemic infections, in which the potentially causal intraoral odontogenic foci often lack acute symptoms. If other origins of infection are not detected, elimination of the potentially causal odontogenic foci should be performed. However, the decision making criteria to eliminate suspected causal teeth is needed to be elucidated through more studies.

## Zusammenfassung

**Einleitung:** Odontogene Infektionsherde können über die lokal korrespondierenden anatomischen Strukturen hinaus durch hämatogene Ausbreitung verschiedene lokale oder generalisierte Infektionen verursachen. Hierbei werden intrakranielle Abszesse am häufigsten beschrieben. Nur wenige Fälle einer extrakraniellen Manifestation von oralen Infektionen werden in der Literatur beschrieben.

**Fallbeschreibung:** Ein 52-jähriger kaukasischer Patient wurde in unserer Klinik mit schwerer Sepsis bei submandibulärem Abszess vorgestellt. Sowohl in der Blutkultur als auch im Erregerabstrich während der chirurgischen Abszessinzision ließ sich *Eggerthia catenaformis* nachweisen, ein Gram-positives anaerobes Stäbchenbakterium, welches dafür bekannt ist, gelegentlich dentogene Abszesse und Bakteriämien zu verursachen. Der Patient entwickelte im weiteren Verlauf einen perihepatischen Abszess mit Kolonperforation, was mehrere chirurgische Interventionen und intravenöse Antibiotikatherapie notwendig machte. Die gesamte stationäre Verweildauer betrug 66 Tage. Fünf Monate nach Entlassung stellte sich der Patient mit Actinomyces-assoziierter Unterkieferosteomyelitis links vor, die ebenfalls chirurgisch therapiert wurde. Drei Jahre nach diesem Eingriff zeigt sich der Patient beschwerdefrei ohne Anhalt auf ein erneutes Auftreten.

**Diskussion:** Insbesondere Mund-, Kiefer-, Gesichtschirurgen sollten den Pathomechanismus von lokalisierten oder generalisierten Infektionen verstehen, da die häufig verursachenden odontogenen Foci nicht immer mit akuter Symptomatik vergesellschaftet sind. Sollte kein anderer wahrscheinlicher Infektfocus gefunden werden, muss jeder potentielle odontogene Fokus saniert werden. Anhand welcher Kriterien und in welchem Umfang diese Sanierung stattzufinden hat, sollte in weiterführenden Studien untersucht werden.

## Background

Odontogenic foci can frequently result in complicated generalized infections and be life-threatening [1]. Most common are intracranial abscesses initiated by oral infections. It is a polymicrobial infection commonly caused by organisms such as microaerophilic streptococci, anaerobic bacteria, *Staphylococcus aureus* and facultative anaerobic gram-negative bacteria [2]. There are four possible routes of odontogenic spread indicated:

1. systemic bacteremia (hematogenous);
2. direct venous drainage via the facial and pterygoid vein systems to the cavernous sinus;
3. inoculation via contiguous extension or by introduction of foreign objects;
4. lymphatic drainage [3].

If direct venous drainage played a predominant role in intracranial dissemination, the incidence of brain infection caused by odontogenic foci would likely be much higher. Therefore, hematogenous spread is considered to be the most important pathophysiological mechanism [3].

Direct contamination results from surgical interventions or trauma and occurs in 10–20% of cases [1]. Furthermore, the orofacial vein circulation is valveless and connects the face, nasal cavity, orbit and paranasal sinuses with the interior of the neurocranium [1]. Thus, the bloodstream could disseminate infections to the central nervous system, a situation that occurs in 20–30% of cases [1]. Dissemination through anatomical cavities, which applies in almost 40% of cases, is also an important factor where the oral cavity is concerned due to the proximity of the nasal cavity and the maxillary sinus [1]. Therefore, dental infections can be the origin of brain infections. In general, the infection could spread from the oral cavity to the ethmoid sinus, the orbital cavity and the brain. 

Besides the brain abscesses, there are also few reports in the international literature regarding generalized infections from primary infections of dental origin in other body parts followed by septic bacteremia and shock symptoms [1], [2], [3]. Different microorganisms such as *Pseudomonas aeruginosa* have been described in the microbiological culture. *Eggerthia catenaformis* is a Gram-positive, anaerobic, non-spore-forming rod that was first isolated from human stools in 1935 and later from intestinal and pleural infections [4], [5]. Based on 16S rRNA gene sequence, *Eggerthia catenaformis* was reclassified in 2011 from *Lactobacillus catenaformis* [5]. The first case of *Eggerthia catenaformis* isolated from the blood cultures of a patient suffering from bacteremia associated with a dental abscess was reported from Kordjian et al. in 2015 [6]. The bacteremia caused by Eggerthia superinfection at that case was successfully treated with intravenous application of benzylpenicillin and metronidazole, to which the isolate was susceptible [6]. In year 2017, Akashi et al. described in their report a severe brain abscess caused by a dental focus [3]. The microbiological examination of the abscess material detected E*ggerthia catenaformis* and the patient was cured after administration of appropriate antibiotic treatment and removal of the odontogenic focus. One year later, Duport et al. published the third case of blood contamination with *Eggerthia catenaformis* caused by odontogenic focus, even though with severe complications such as pleural empyema and pulmonary abscess [7]. 

Here, we present a new unusual case of infection with *Eggerthia catenaformis* initiated from a submandibular abscess on the left side of posterior mandible and resulting perihepatic abscess and severe secondary abdominal infection. This is the fourth case of odontogenic infection with *Eggerthia catenaformis* described in the literature. 

Aim of this article is also to alert oral and maxillofacial surgeons as well as the dental professional community to understand the pathomechanism of severe general complications with septic symptomatology possibly caused by odontogenic foci and provide a literature review of oral infections with *Eggerthia catenaformis*. 

## Case description

A 52-year-old Caucasian male patient presented to our craniomaxillofacial surgery department (Ludwigshafen hospital in Germany) with an extended submandibular abscess on the left side and symptoms of generalized sepsis. 

The patient’s general medical history revealed obesity (body mass index of 42), smoking history (30 pack years) and chronic alcoholism. Further comorbidities included arterial hypertension treated with appropriate medication. 

An intraoral clinical examination revealed severe caries of the first molars on the left and right mandible (teeth #36 and #46) with signs of mucosal abscess or purulent discharge. Moderate marginal periodontitis of the remaining teeth was also diagnosed, with 2 mm attachment loss, 2–3 mm probing depth, and a horizontal bony defect. Caries lesions and generalized periodontitis were also diagnosed radiologically. The patient mentioned also difficulty of swallowing. Mouth opening was reduced at 1.5 cm. Extraorally, an extended submandibular abscess at the left side was detected. 

Subsequently, the patient underwent surgical treatment under general anesthesia, which consisted of extraoral incision and drainage of the submandibular abscess and the removal of the lower first right molars of both sides. The intravenous admission of antibiotics with ampicillin/sulbactam 3 gr (Unacid^®^ , Pfizer Pharma GmbH, Germany) three times a day and metronidazol 500 mg (Clont^®^, INFECTOPHARM Arzneimittel and Consilium GmbH, Germany) two times a day combined with appropriate analgesia was applied under hospitalization. The postoperative surveillance occurred at the intensive care unit of our hospital for further two days. The transfusion of one erythrocytes concentrate was essential by a haemoglobin level of 6.2 g/dl. A nasogastric tube was used for continuous feeding during this period to avoid soft tissue manipulation at the surgical sites. Once the patient was stabilized and his infection blood parameters were reduced within two days (leucocytes: 12.2/nl to 11.1/nl; c-reactive protein: 310.4 to 283.5), the patient was transferred to the craniomaxillofacial ward for further treatment and monitoring. During the time of hospital admission, the swelling was treated extraorally with an antiseptic solution (Octenisept^®^, Schülke & Mayr GmbH, Germany) under continuous antibiotic therapy.

Microbiological examination of the abscess fluid and an additional blood culture identified *Eggerthia catenaformis* as the pathogen. *Eggerthia catenaformis* was confirmed by MALDI-TOF mass spectrometry (Bruker Daltronik, Bremen, Germany), and antibiotic treatment was changed to piperacilline/tazobactam 4/0.5 gr combination (Tazobac^®^, Pfizer Pharma GmbH, Germany) four times a day accordingly.

**Figure 1:**
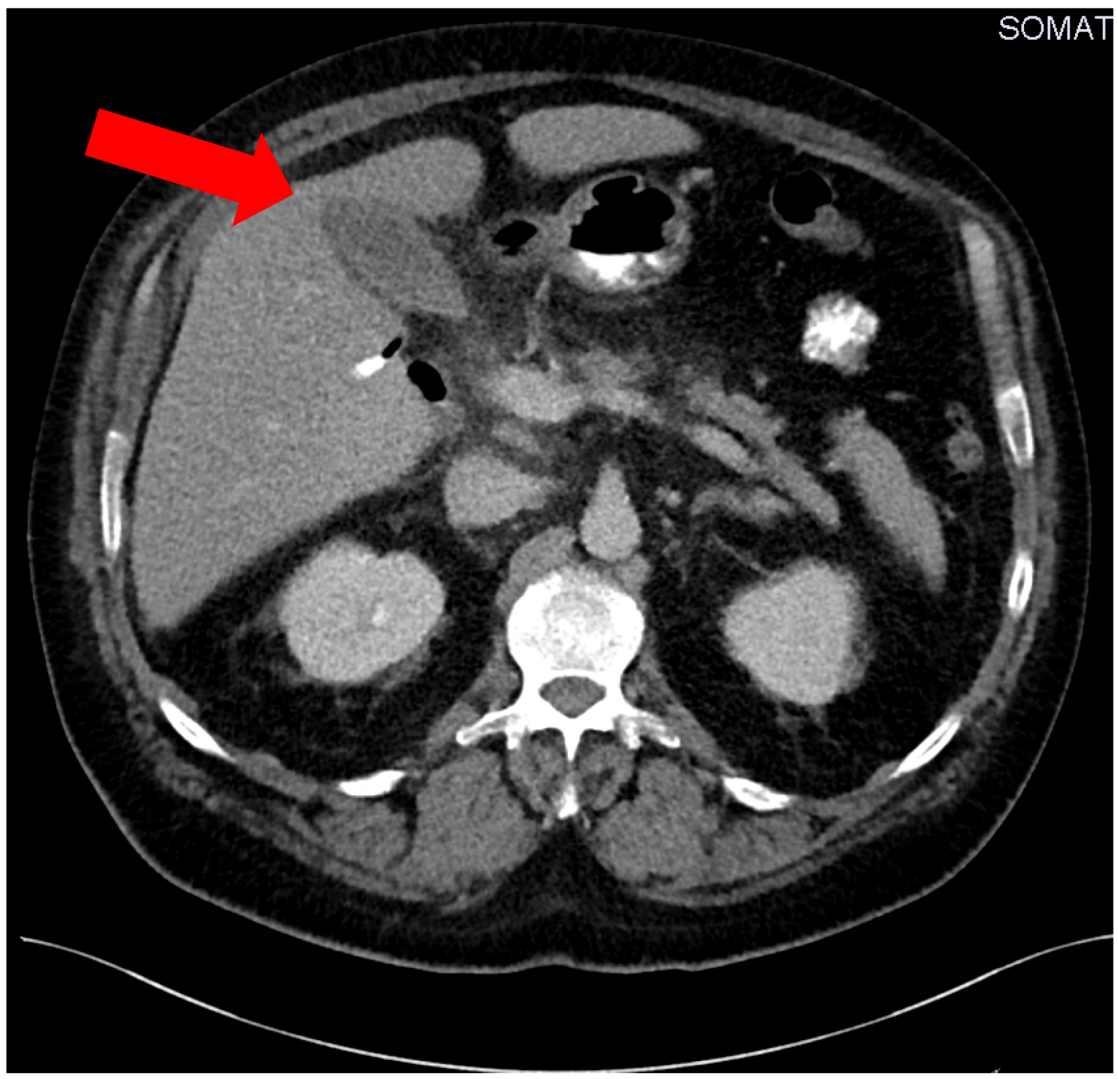
Preoperative computed tomography of the hepatic abscess (red arrow)

In the following days the patient’s situation destabilized showing clinical signs and symptoms auf acute abdomen. An abdominal CT scan on the ninth day after initial admission revealed a perihepatic abscess (Figure 1 (Fig. 1)). This was treated with immediate surgical intervention and drainage of the hepatic abscess by visceral surgeons of our hospital. An additional microbiological examination of the hepatic abscess material confirmed the contamination with *Eggerthia catenaformis*. Postoperatively, the patient received another three erythrocyte concentrate transfusions (500 ml each) and 2000 IE prothrombin complex to treat persistent anaemia and ethyl-toxic blood coagulation disorder.

One week after the hepatic drainage, the patient developed an abdominal wound infection, which progressed to an open abdomen. This was treated by inserting a vicryl net and several vacuum seals which were changed every day. Thirty days after the open abdomen was diagnosed, *Staphylococcus aureus* and *Finegoldia magna* were identified as the pathogens responsible for the open abdominal wound infection. *Eggerthia* *catenaformis* was no longer detected in the abdominal wound liquid and the blood culture. Seventeen days later, *Staphylococcus aureus* was only detected in the microbiological examination without traces of *Finegoldia* *magna* or *Eggerthia catenaformis* remained. Sixty-six days after initial admission, the patient was dismissed from our hospital with stable blood parameters and without signs of active infection or wound dehiscences.

**Figure 2:**
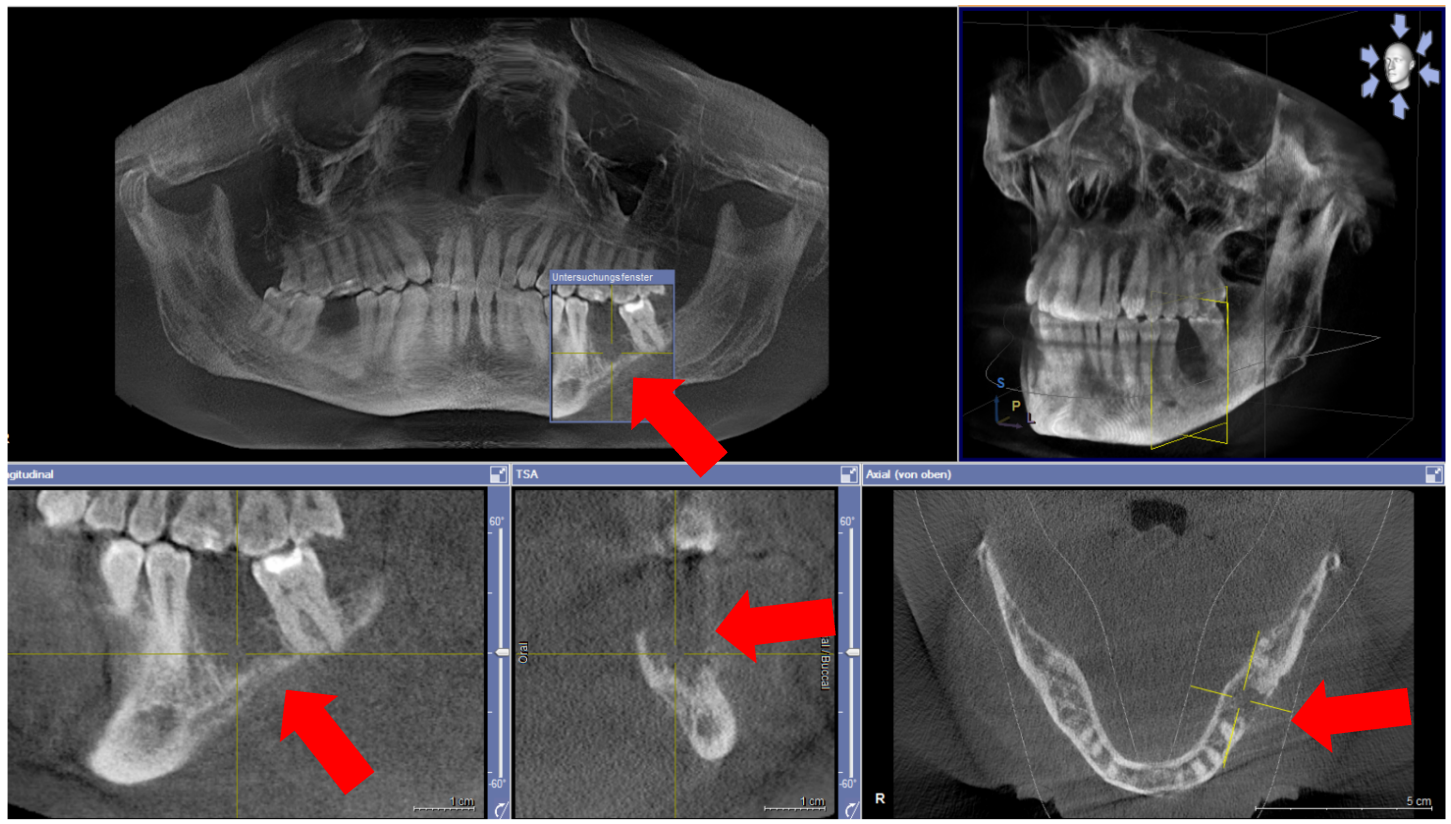
Cone Beam Computed Tomography (CBCT) with display of subsequent osteonecrosis of the lower left mandible in region 036 (red arrows)

Five months after dismission, the patient was re-admitted to the craniomaxillofacial department with exposed mandibular bone in the area of the extracted first left molar and recurring pain (Figure 2 (Fig. 2)). No signs of mucosal abscess or purulent discharge were detected. The clinical and radiographic examination revealed mandibular osteonecrosis and the patient underwent surgery under general anesthesia after 24 hours of preoperative intravenous antibiotic treatment under hospitalization. This involved partial decortication of the infected mandible region (the residual anterior-posterior alveolar process and the bicortical plate exposed to vital bone) with preservation of the alveolar inferior nerve and the basal cortical margin. After resection, residual infected and necrotic tissues were removed from the remaining avital bone surface by a rotating burr and diamond burr in an attempt to prevent recurrence of osteomyelitis. The wound was closed after periosteal releasing incisions to achieve a tension-free adaption of the soft tissues. As described earlier, an adjuvant cycle of antibiotics was administered and a nasogastric tube was fitted under hospitalization for further 7 days. The local treatment consisted of irrigating the mouth with chlorhexidine and appropriate mouth hygiene. After hospitalization, no more antibiotics were administered. On review, there was a considerable improvement in the patient’s physical condition; the systemic symptoms had objectively subsided and the pain reduced, decreasing the analgesic intake. Two weeks post-operation, we noticed a complete remission of the clinical symptoms, without further dehiscence of the alveolar bone and the soft tissues. A postoperative orthopantomograph was performed 3 months after surgical decortication showed sufficient alveolar bone healing without radiologic signs of osteonecrosis.

The surgical specimen was fixed in neutral-buffered formalin and sent to the pathological anatomy department of our hospital, where it was decalcified in formic acid, embedded in paraffin, sectioned at 4-µm thickness, and stained with hematoxylin-eosin. The histopathological analysis of the decalcified samples showed areas of bone necrosis with inflammatory cell infiltration, empty Haversian canals and non-vital bone with a superinfection of Actinomyces and partial florid osteitis, thus confirming the clinical diagnosis of jaw osteomyelitis. 

At the time of this report, 3 years after the initial *Eggerthia catenaformis* infection, no further infections, complications or symptoms of recurrence have occurred. 

## Discussion

The aim of this article was to report an original case of *Eggerthia catenaformis* infection initiated from a submandibular abscess and causing perihepatic abscess and severe clinical sepsis.

*Eggerthia catenaformis*, whose natural habitat is human feces and can occur throughout the gastro-intestinal system, has rarely been reported as a human pathogen [5], [6], [7]. As it has been previously isolated from several infection sites, and its genome seems primed for a pathogenic interaction with the host, it should be monitored carefully as a possible emerging human pathogen. It is envisaged that this second draft genome sequence of *Eggerthia catenaformis* will be useful in determining the potential pathogenicity as well as antimicrobial resistance capabilities of future isolates [5].

Only three clinical cases of *Eggerthia catenaformis* infections have been described to date [3], [6], [7]. Two cases presented with a brain abscess caused by oral infection, the third one with pleural empyema and pulmonary abscess. In the report presented by Kordjian et al., MIC analysis showed that the *Eggerthia catenaformis* strain was sensitive to penicillin, clindamycin, metronidazole, meropenem, moxifloxacin, and vancomycin (with a low MIC value) [6]. Kordjian et al. [6] also demonstrated the value of MALDI-TOF MS for identification of *Eggerthia catenaformis*. In the clinical case of Duport et al. [7], the isolated *Eggerthia* *catenaformis* strain was multisensitive, but it had a moderately high MIC value for metronidazole (0.75 mg/L). The best MIC value was for penicillin. This finding highlights the importance of rapidly treating with penicillin after identification of the microorganism. 

The affected patients in the three case reports mentioned above were not predisposed with underlying medical conditions but still had developed a polymicrobial infection. In contrast to these patients, the patient’s immunological system in our report was already weakened due obesity, previous chronic alcohol abuse and smoking habit. The ability of adequate response to systemic infections is insufficient by patients with underlying medical conditions such chronic pulmonary and cardiologic diseases, diabetes mellitus, severe obesity or an immune-deficiency due to cancer, malnutrition, certain genetic disorders or HIV infection. Additionally, certain pharmacological treatments and other therapies such as antiresorptive medication, radiation therapy and stem cell transplantation as well as exogenic habits such as smoking and alcohol consume could affect negatively the immunological system. This kind of predisposing factors could increase the risk of developing such kind of systemic infection spread. 

Our main hypothesis is that our patient developed a perihepatic infection via hematogenous spread secondary to the dental abscess. Although, this could have been also facilitated due the possible hepatic damage, already predisposed from the chronic alcohol consume and obesity. The patient recovered after a combination of surgical interventions and treatment with prolonged intravenous antibiotics. The germ was susceptible to penicillin und β-lactamase inhibitors combined with metronidazol. 

Given the polymicrobial nature of the infection in all four cases, the pathogenicity of *Eggerthia catenaformis* should be discussed. The gold standard for identifying *Eggerthia catenaformis* is MALDI-TOF mass spectrometry [6]. Recently, a second genome sequence of *Eggerthia catenaformis* has led to a better appreciation of the bacterium’s pathogenicity and resistance profile [5], [7], [8]. 

Summarizing, this is the fourth report of an *Eggerthia catenaformis* infection with dental origin followed by general septic condition. This bacterium seems to be capable of causing very severe infections, especially in immunocompromised patients. It may also cause secondary osteonecrosis of the jaw. Thus, our clinical case and those previously reported propose the need of close clinical monitoring and extended antibiotic treatment, combined with rapid surgical intervention, when *Eggerthia catenaformis* bacteremia is present [7]. The purpose is to prevent severe bacteremia and further organ contamination. 

## Conclusion

*Eggerthia catenaformis* may be responsible for severe infections in association with other microorganisms or predisposing health factors. This kind of infection should be treated with penicillin/ß-lactamase inhibitors in combination with metronidazol, and their presence should alert for the detection and rapid treatment of the possible dental focus. Reporting infections caused by uncommon pathogens such as *Eggerthia catenaformis* and analyzing the MICs of antibacterial agents is necessary to determine optimal antibiotic regimens in different settings. 

Oral and maxillofacial surgeons and dental practitioners should understand the characteristics of severe infections such as brain abscess, sepsis signs and/or general disorders, in which potentially causal odontogenic focus often lack acute symptoms. If other origins of infection such as sinusitis and otitis media are absent, it is suggested to eliminate the potentially causal dental focus in order to improve oral hygiene and prevent bacteremic spread. The decision making criteria to eliminate suspected causal teeth should be considered accordingly to the guidelines of dental management for patients undergoing cardiac valve surgery or bisphosphonate medication.

## Notes

### Ethical statement

This research was conducted in full accordance with ethical principles, including the World Medical Association Declaration of Helsinki. The patient’s data was referenced to with the understanding and written consent of the patient, and all data was also anonymized and de-identified prior to analysis. 

### Competing interests

The authors declare that they have no competing or financial interests, either directly or indirectly, in the products listed in the study.
